# Supplementary material for: Chalcogen Noncovalent Interactions between Diazines and Sulfur Oxides in Supramolecular Circular Chains
Source: Int J Mol Sci. 2024 Jul 8;25(13):7497. doi: 10.3390/ijms25137497 (PMC11242197; doi:10.3390/ijms25137497)
Supplement: Supplementary file 1 [file ijms-25-07497-s001.zip › ijms-3080932-supplementary.pdf]

SUPPORTING INFORMATION

Chalcogen Noncovalent Interactions between Diazines and Sulfur Oxides in Supramolecular Circular Chains

Emna Rahali, Zahra Noori, Youssef Arfaoui and Jordi Poater

Contents

Figure S1. Molecular electrostatic potential isosurface (electronic density isovalue = 0.03 a.u.) of complexes of SO_2 with pyridazine, pyrimidine, pyrazine with 3 and 6 units.

Figure S2. Molecular electrostatic potential isosurface (electronic density isovalue = 0.03 a.u.) of complexes of SO_3 with pyridazine, pyrimidine, pyrazine with 3units, together with SO_3 and its VDD charges (in milli-au).

Figure S3. Fukui plus (top) and Fukui minus (bottom) functions of the diazines and SO_2/SO_3 systems under analysis (isovalue = 0.003).

Table S1. Energy decomposition analysis (in kcal mol^{-1}) divided by the number of molecules for each system (2n) of the SO_2 complexes with pyridazine, pyrimidine and pyrazine.

Table S2. Cartesian coordinates of all systems under analysis together with ADF total bonding energy (in kcal mol^{-1}, in parentheses).

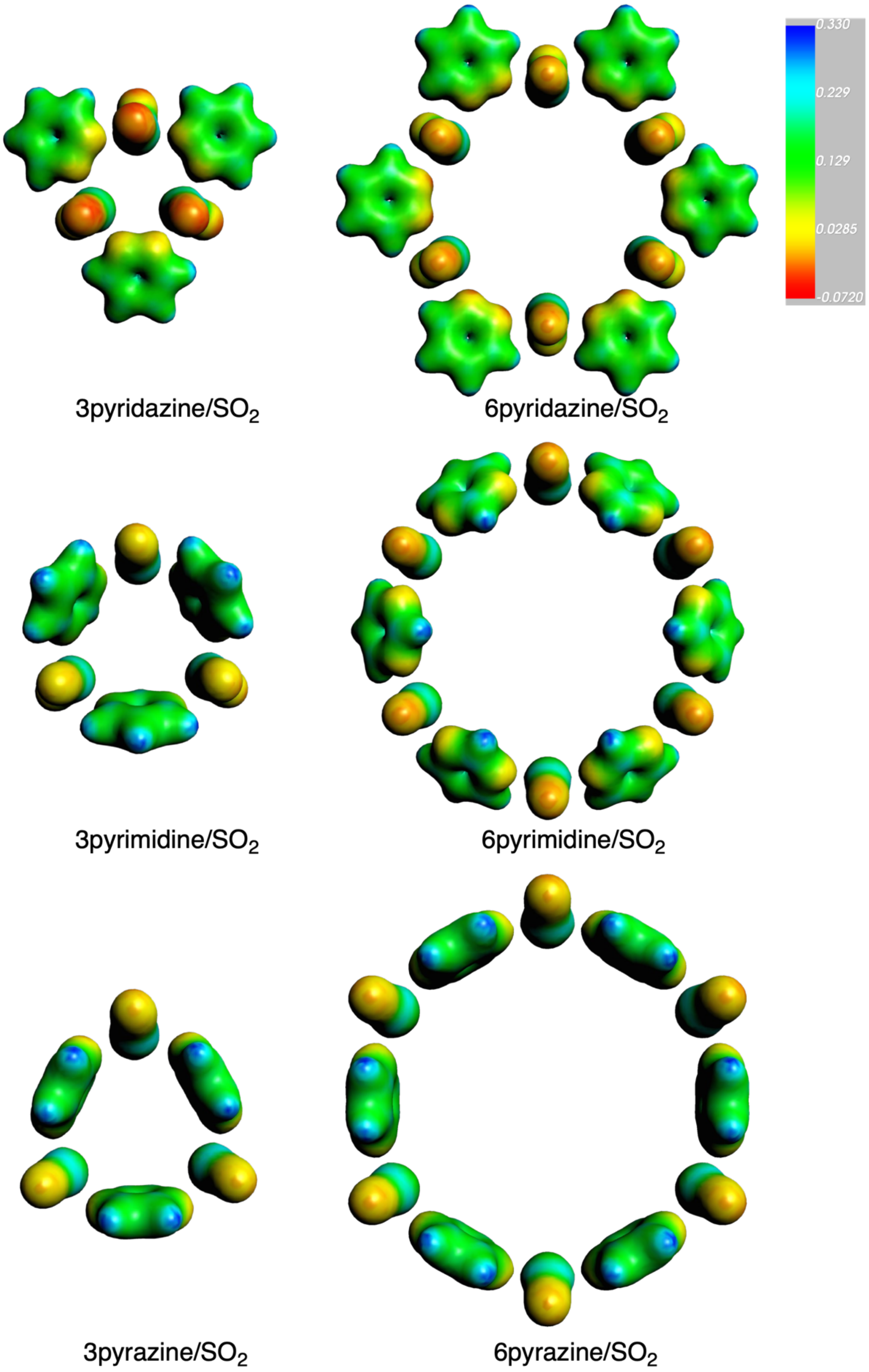

Figure S1. Molecular electrostatic potential isosurface (electronic density isovalue = 0.03 a.u.) of complexes of SO_2 with pyridazine, pyrimidine, pyrazine with 3 and 6 units.

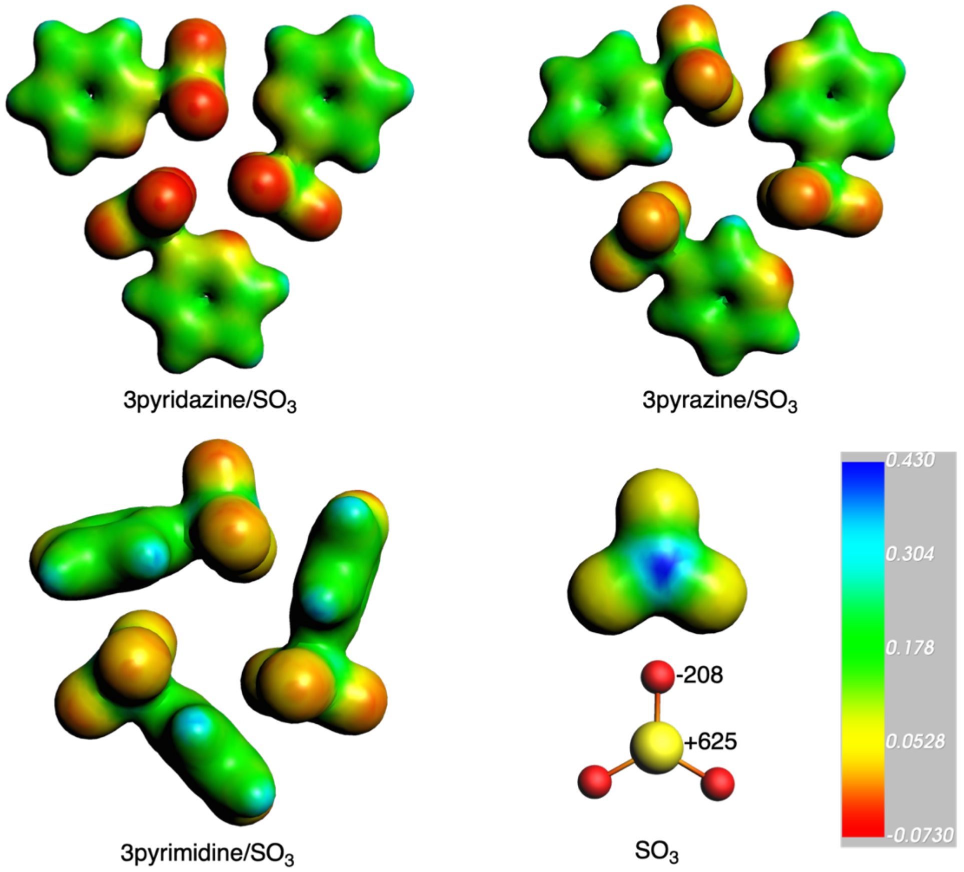

Figure S2. Molecular electrostatic potential isosurface (electronic density isovalue = 0.03 a.u.) of complexes of SO_3 with pyridazine, pyrimidine, pyrazine with 3units, together with SO_3 and its VDD charges (in milli-au).

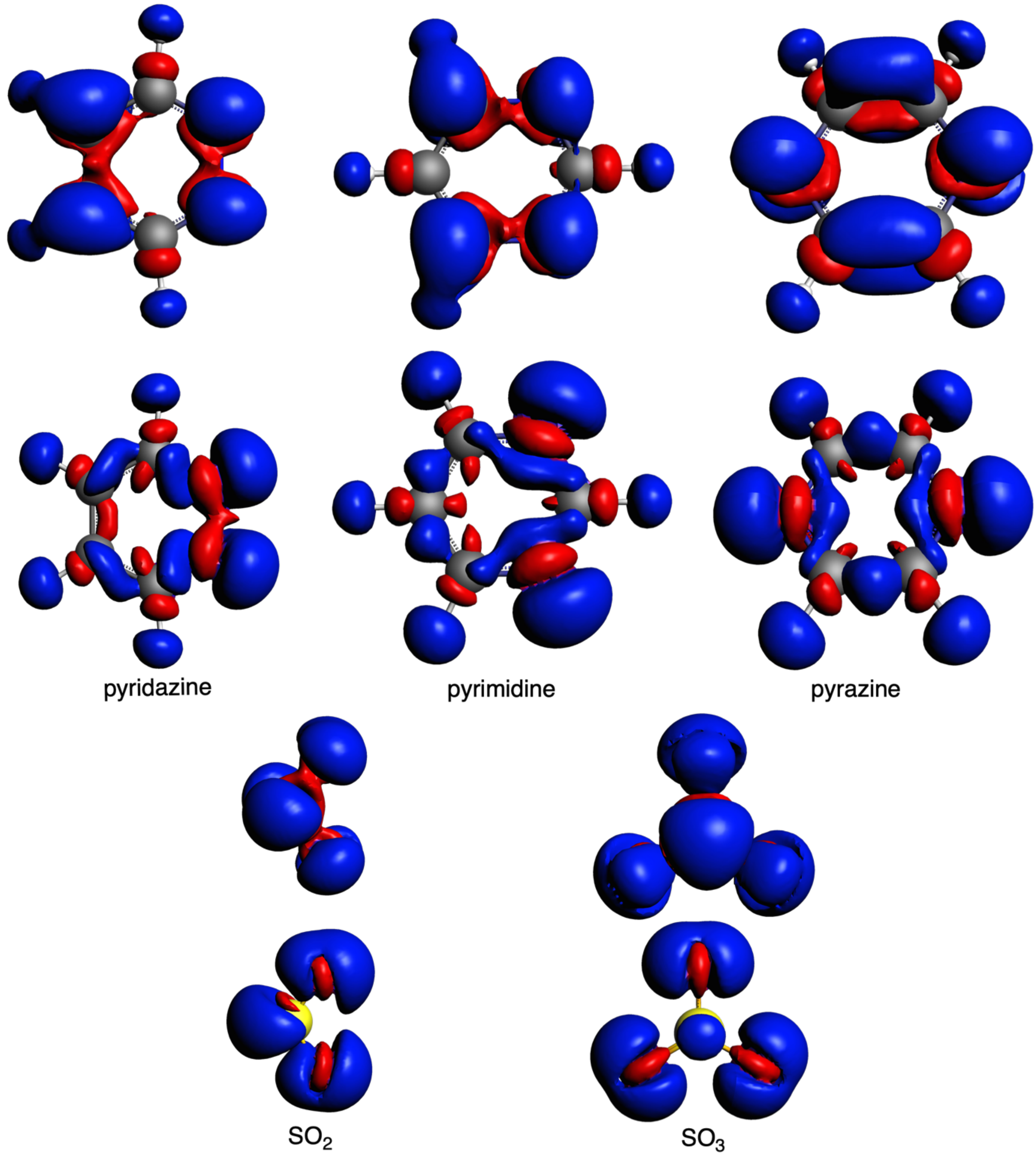

Figure S3. Fukui plus (top) and Fukui minus (bottom) functions of the diazines under analysis (isovalue = 0.003).

Table S1. Energy decomposition analysis (in kcal mol^{-1}) divided by the number of molecules for each system (2n) of the SO_2 complexes with pyridazine, pyrimidine and pyrazine.[a]

	ΔE_{int}	ΔE_{Pauli}	ΔV_{elstat}	ΔE_{oi}	ΔE_{disp}
					**pyridazine**
**3**	-8.1	19.4	-14.2	-9.2	-4.1
**4**	-8.1	17.9	-13.2	-8.7	-4.1
**5**	-7.5	14.4	-10.9	-7.0	-3.9
**6**	-6.9	12.7	-9.7	-5.9	-4.0
					**pyrimidine**
**3**	-6.5	14.4	-10.4	-6.6	-3.9
**4**	-7.1	16.4	-11.9	-7.7	-3.9
**5**	-7.5	17.8	-13.0	-8.4	-3.9
**6**	-7.7	18.5	-13.5	-8.7	-4.0
					**pyrazine**
**3**	-5.7	14.3	-9.7	-6.4	-3.8
**4**	-6.3	15.9	-11.2	-7.2	-3.8
**5**	-6.6	17.9	-12.6	-8.0	-3.9
**6**	-6.7	19.1	-13.4	-8.5	-3.9

[a] Computed at BLYP-D3(BJ)/TZ2P level of theory. $\Delta E_{int} = \Delta E_{Pauli} + \Delta V_{elstat} + \Delta E_{oi} + \Delta E_{disp}$.

Table S2. Cartesian coordinates of all systems under analysis together with ADF total bonding energy (in kcal mol^{-1}, in parentheses).

SO2 (-369.2)

1	S	0.00000000	0.00000000	-0.49332231
2	O	0.00000000	1.26307942	0.24666115
3	O	-0.00000000	-1.26307942	0.24666115

Pyridazine (-1447.6)

1	H	-0.00000000	-1.27268473	-1.73645540
2	N	0.00000000	0.67387623	1.60532402
3	N	-0.00000000	-0.67387623	1.60532402
4	C	-0.00000000	1.32920836	0.43232669
5	C	0.00000000	-1.32920836	0.43232669
6	C	0.00000000	-0.69386114	-0.81574004
7	C	-0.00000000	0.69386114	-0.81574004
8	H	-0.00000000	2.41535178	0.51454473
9	H	0.00000000	-2.41535178	0.51454473
10	H	0.00000000	1.27268473	-1.73645540

2pyridazine/SO2 (-3658.2)

1	C	3.73913148	-1.33557828	0.01061238
2	C	3.73913148	1.33557828	0.01061238
3	C	4.92694265	0.69391848	-0.36304399
4	C	4.92694265	-0.69391848	-0.36304399
5	H	3.65091638	-2.42033047	0.02984551
6	H	3.65091638	2.42033047	0.02984551
7	H	5.80454418	1.27125247	-0.64267315
8	H	5.80454418	-1.27125247	-0.64267315
9	O	0.00000000	2.75951409	1.60137260
10	O	0.00000000	2.39757529	-0.87551064
11	S	0.00000000	1.81226964	0.47550778
12	N	2.63220349	0.66904839	0.36457438
13	N	2.63220349	-0.66904839	0.36457438
14	S	-0.00000000	-1.81226964	0.47550778
15	O	-0.00000000	-2.75951409	1.60137260
16	O	-0.00000000	-2.39757529	-0.87551064
17	C	-3.73913148	-1.33557828	0.01061238
18	C	-3.73913148	1.33557828	0.01061238
19	C	-4.92694265	-0.69391848	-0.36304399
20	H	-3.65091638	-2.42033047	0.02984551
21	C	-4.92694265	0.69391848	-0.36304399
22	H	-3.65091638	2.42033047	0.02984551
23	H	-5.80454418	-1.27125247	-0.64267315
24	H	-5.80454418	1.27125247	-0.64267315
25	N	-2.63220349	-0.66904839	0.36457438
26	N	-2.63220349	0.66904839	0.36457438

3pyridazine/SO2 (-5496.3)

1	C	3.46164224	3.31657358	-0.11519200
2	C	1.14141585	4.65615691	-0.11519200
3	C	2.30995966	5.38990083	-0.35522464
4	C	3.51281121	4.69543416	-0.35522464
5	H	4.34773419	2.68358453	-0.11143104
6	H	0.15018529	5.10704053	-0.11143104
7	H	2.26418646	6.46070347	-0.53678879
8	H	4.46304010	5.19119472	-0.53678879
9	O	-1.46862382	-2.54373109	1.92491791
10	O	-1.90709116	-3.30317878	-0.41274945

11	N	1.15917828	3.33823632	0.11939038
12	N	2.31140831	2.67299600	0.11939038
13	O	2.93724765	-0.00000000	1.92491791
14	C	3.46164224	-3.31657358	-0.11519200
15	C	1.14141585	-4.65615691	-0.11519200
16	C	3.51281121	-4.69543416	-0.35522464
17	H	4.34773419	-2.68358453	-0.11143104
18	C	2.30995966	-5.38990083	-0.35522464
19	H	0.15018529	-5.10704053	-0.11143104
20	H	4.46304010	-5.19119472	-0.53678879
21	H	2.26418646	-6.46070347	-0.53678879
22	N	2.31140831	-2.67299600	0.11939038
23	N	1.15917828	-3.33823632	0.11939038
24	S	-1.31968864	2.28576777	0.48632370
25	O	-1.46862382	2.54373109	1.92491791
26	O	-1.90709116	3.30317878	-0.41274945
27	S	2.63937727	-0.00000000	0.48632370
28	S	-1.31968864	-2.28576777	0.48632370
29	C	-4.60305809	-1.33958333	-0.11519200
30	C	-5.82277087	-0.69446667	-0.35522464
31	H	-4.49791948	-2.42345599	-0.11143104
32	C	-4.60305809	1.33958333	-0.11519200
33	C	-5.82277087	0.69446667	-0.35522464
34	H	-6.72722656	-1.26950875	-0.53678879
35	H	-4.49791948	2.42345599	-0.11143104
36	H	-6.72722656	1.26950875	-0.53678879
37	N	-3.47058659	-0.66524032	0.11939038
38	N	-3.47058659	0.66524032	0.11939038
39	O	3.81418231	-0.00000000	-0.41274945

4pyridazine/SO2 (-7328.7)

1	C	5.69534913	-1.33784610	0.16113778
2	C	6.92531250	-0.69408554	0.34284090
3	H	5.59237430	-2.42146294	0.15367488
4	C	5.69534913	1.33784610	0.16113778
5	C	6.92531250	0.69408554	0.34284090
6	H	7.83699267	-1.27057475	0.47842380
7	H	5.59237430	2.42146294	0.15367488
8	H	7.83699267	1.27057475	0.47842380
9	C	1.33784610	5.69534913	0.16113778
10	C	0.69408554	6.92531250	0.34284090
11	H	2.42146294	5.59237430	0.15367488
12	C	-1.33784610	5.69534913	0.16113778
13	C	-0.69408554	6.92531250	0.34284090
14	H	1.27057475	7.83699267	0.47842380
15	H	-2.42146294	5.59237430	0.15367488
16	H	-1.27057475	7.83699267	0.47842380
17	C	-5.69534913	1.33784610	0.16113778
18	C	-5.69534913	-1.33784610	0.16113778
19	C	-6.92531250	0.69408554	0.34284090
20	H	-5.59237430	2.42146294	0.15367488
21	C	-6.92531250	-0.69408554	0.34284090
22	H	-5.59237430	-2.42146294	0.15367488
23	H	-7.83699267	1.27057475	0.47842380
24	H	-7.83699267	-1.27057475	0.47842380
25	C	-1.33784610	-5.69534913	0.16113778
26	C	-0.69408554	-6.92531250	0.34284090
27	H	-2.42146294	-5.59237430	0.15367488
28	C	1.33784610	-5.69534913	0.16113778
29	C	0.69408554	-6.92531250	0.34284090

30	H	-1.27057475	-7.83699267	0.47842380
31	H	2.42146294	-5.59237430	0.15367488
32	H	1.27057475	-7.83699267	0.47842380
33	N	0.66666112	-4.54871932	-0.01220674
34	N	-0.66666112	-4.54871932	-0.01220674
35	N	-4.54871932	-0.66666112	-0.01220674
36	N	-4.54871932	0.66666112	-0.01220674
37	N	-0.66666112	4.54871932	-0.01220674
38	N	0.66666112	4.54871932	-0.01220674
39	N	4.54871932	0.66666112	-0.01220674
40	N	4.54871932	-0.66666112	-0.01220674
41	O	3.54371151	3.54371151	0.15986669
42	O	2.58112968	2.58112968	-1.93702557
43	O	2.58112968	-2.58112968	-1.93702557
44	O	3.54371151	-3.54371151	0.15986669
45	O	-3.54371151	-3.54371151	0.15986669
46	O	-2.58112968	-2.58112968	-1.93702557
47	O	-2.58112968	2.58112968	-1.93702557
48	O	-3.54371151	3.54371151	0.15986669
49	S	2.59603160	2.59603160	-0.47058236
50	S	2.59603160	-2.59603160	-0.47058236
51	S	-2.59603160	-2.59603160	-0.47058236
52	S	-2.59603160	2.59603160	-0.47058236

5pyridazine/SO2 (-9155.7)

1	C	-6.96264136	1.33441967	-0.13053530
2	C	-6.96264136	-1.33441967	-0.13053530
3	C	-8.20672179	0.69332596	-0.10584665
4	H	-6.86772668	2.41811195	-0.14280983
5	C	-8.20672179	-0.69332596	-0.10584665
6	H	-6.86772668	-2.41811195	-0.14280983
7	H	-9.12655051	1.27261684	-0.08894971
8	H	-9.12655051	-1.27261684	-0.08894971
9	C	-0.88246599	7.03422379	-0.13053530
10	C	-1.87662433	8.01930574	-0.10584665
11	H	0.17751687	7.27883391	-0.14280983
12	C	-3.42068304	6.20950708	-0.13053530
13	C	-3.19540868	7.59080674	-0.10584665
14	H	-1.60992867	9.07312556	-0.08894971
15	H	-4.42200538	5.78435853	-0.14280983
16	H	-4.03058975	8.28660509	-0.08894971
17	C	6.41724739	3.01296972	-0.13053530
18	C	7.04690417	4.26287756	-0.10584665
19	H	6.97743815	2.08045480	-0.14280983
20	C	4.84854298	5.17210610	-0.13053530
21	C	6.23185063	5.38470253	-0.10584665
22	H	8.13155987	4.33488314	-0.08894971
23	H	4.13477706	5.99304212	-0.14280983
24	H	6.63550905	6.39402044	-0.08894971
25	C	4.84854298	-5.17210610	-0.13053530
26	C	6.23185063	-5.38470253	-0.10584665
27	H	4.13477706	-5.99304212	-0.14280983
28	C	6.41724739	-3.01296972	-0.13053530
29	C	7.04690417	-4.26287756	-0.10584665
30	H	6.63550905	-6.39402044	-0.08894971
31	H	6.97743815	-2.08045480	-0.14280983
32	H	8.13155987	-4.33488314	-0.08894971
33	C	-3.42068304	-6.20950708	-0.13053530
34	C	-0.88246599	-7.03422379	-0.13053530
35	C	-3.19540868	-7.59080674	-0.10584665

36	H	-4.42200538	-5.78435853	-0.14280983
37	C	-1.87662433	-8.01930574	-0.10584665
38	H	0.17751687	-7.27883391	-0.14280983
39	H	-4.03058975	-8.28660509	-0.08894971
40	H	-1.60992867	-9.07312556	-0.08894971
41	N	5.08475879	2.86798697	-0.14493571
42	N	4.29889457	3.94963627	-0.14493571
43	N	-1.15634081	5.72214970	-0.14493571
44	N	-2.42789583	5.30899642	-0.14493571
45	N	-5.79941672	0.66849603	-0.14493571
46	N	-5.79941672	-0.66849603	-0.14493571
47	N	-2.42789583	-5.30899642	-0.14493571
48	N	-1.15634081	-5.72214970	-0.14493571
49	N	4.29889457	-3.94963627	-0.14493571
50	N	5.08475879	-2.86798697	-0.14493571
51	S	1.49614704	-4.60466714	0.13633791
52	S	-3.91696383	-2.84584080	0.13633791
53	S	-3.91696383	2.84584080	0.13633791
54	S	1.49614704	4.60466714	0.13633791
55	S	4.84163355	0.00000000	0.13633791
56	O	-3.90913639	-2.84015383	1.60236757
57	O	-4.99215748	-3.62701472	-0.51255110
58	O	-4.99215748	3.62701472	-0.51255110
59	O	-3.90913639	2.84015383	1.60236757
60	O	1.90683448	5.86863309	-0.51255110
61	O	1.49315723	4.59546544	1.60236757
62	O	4.83195830	0.00000000	1.60236757
63	O	6.17064600	0.00000000	-0.51255110
64	O	1.49315723	-4.59546544	1.60236757
65	O	1.90683448	-5.86863309	-0.51255110

6pyridazine/SO2 (-10980.7)

1	C	-5.21930796	6.37372314	-0.06918853
2	C	-2.91015217	7.70691485	-0.06918853
3	C	-5.28353560	7.76508454	0.06746970
4	H	-6.11470256	5.75750173	-0.09405097
5	C	-4.08299268	8.45821833	0.06746970
6	H	-1.92879148	8.17423863	-0.09405097
7	H	-6.24276489	8.26698399	0.16699221
8	H	-4.03803571	9.53988498	0.16699221
9	C	-8.12946013	-1.33319171	-0.06918853
10	C	-9.36652828	-0.69313378	0.06746970
11	H	-8.04349404	-2.41673689	-0.09405097
12	C	-8.12946013	1.33319171	-0.06918853
13	C	-9.36652828	0.69313378	0.06746970
14	H	-10.28080060	-1.27290098	0.16699221
15	H	-8.04349404	2.41673689	-0.09405097
16	H	-10.28080060	1.27290098	0.16699221
17	C	-2.91015217	-7.70691485	-0.06918853
18	C	-4.08299268	-8.45821833	0.06746970
19	H	-1.92879148	-8.17423863	-0.09405097
20	C	-5.21930796	-6.37372314	-0.06918853
21	C	-5.28353560	-7.76508454	0.06746970
22	H	-4.03803571	-9.53988498	0.16699221
23	H	-6.11470256	-5.75750173	-0.09405097
24	H	-6.24276489	-8.26698399	0.16699221
25	C	5.21930796	-6.37372314	-0.06918853
26	C	5.28353560	-7.76508454	0.06746970
27	H	6.11470256	-5.75750173	-0.09405097
28	C	2.91015217	-7.70691485	-0.06918853

29	C	4.08299268	-8.45821833	0.06746970
30	H	6.24276489	-8.26698399	0.16699221
31	H	1.92879148	-8.17423863	-0.09405097
32	H	4.03803571	-9.53988498	0.16699221
33	C	8.12946013	1.33319171	-0.06918853
34	C	9.36652828	0.69313378	0.06746970
35	H	8.04349404	2.41673689	-0.09405097
36	C	8.12946013	-1.33319171	-0.06918853
37	C	9.36652828	-0.69313378	0.06746970
38	H	10.28080060	1.27290098	0.16699221
39	H	8.04349404	-2.41673689	-0.09405097
40	H	10.28080060	-1.27290098	0.16699221
41	C	2.91015217	7.70691485	-0.06918853
42	C	5.21930796	6.37372314	-0.06918853
43	C	4.08299268	8.45821833	0.06746970
44	H	1.92879148	8.17423863	-0.09405097
45	C	5.28353560	7.76508454	0.06746970
46	H	6.11470256	5.75750173	-0.09405097
47	H	4.03803571	9.53988498	0.16699221
48	H	6.24276489	8.26698399	0.16699221
49	O	5.42881880	-3.13433000	1.33582674
50	O	6.20907826	-3.58481301	-0.99662835
51	O	5.42881880	3.13433000	1.33582674
52	O	6.20907826	3.58481301	-0.99662835
53	O	-0.00000000	6.26865999	1.33582674
54	O	0.00000000	7.16962600	-0.99662835
55	O	-5.42881880	3.13433000	1.33582674
56	O	-6.20907826	3.58481301	-0.99662835
57	O	-6.20907826	-3.58481301	-0.99662835
58	O	-5.42881880	-3.13433000	1.33582674
59	O	0.00000000	-6.26865999	1.33582674
60	O	-0.00000000	-7.16962600	-0.99662835
61	S	-0.00000000	-5.99417107	-0.10578078
62	S	5.19110442	-2.99708553	-0.10578078
63	S	5.19110442	2.99708553	-0.10578078
64	S	0.00000000	5.99417107	-0.10578078
65	S	-5.19110442	2.99708553	-0.10578078
66	S	-5.19110442	-2.99708553	-0.10578078
67	N	4.06462502	-5.70179117	-0.18793120
68	N	2.90558350	-6.37096410	-0.18793120
69	N	-2.90558350	-6.37096410	-0.18793120
70	N	-4.06462502	-5.70179117	-0.18793120
71	N	-6.97020850	-0.66917293	-0.18793120
72	N	-6.97020850	0.66917293	-0.18793120
73	N	-4.06462502	5.70179117	-0.18793120
74	N	-2.90558350	6.37096410	-0.18793120
75	N	2.90558350	6.37096410	-0.18793120
76	N	4.06462502	5.70179117	-0.18793120
77	N	6.97020850	0.66917293	-0.18793120
78	N	6.97020850	-0.66917293	-0.18793120

Pyrimidine (-1468.4)

1	C	-3.58709353	3.83378711	0.00000000
2	C	-2.92257743	5.06205260	0.00000000
3	H	-4.67590648	3.77940341	0.00000000
4	C	-1.52661026	5.02340767	0.00000000
5	H	-3.46626797	6.00375223	0.00000000
6	H	-0.93510612	5.93915550	0.00000000
7	N	-2.92654729	2.66185100	0.00000000
8	C	-1.58474240	2.74485435	0.00000000

9	H	-1.03960952	1.80065651	0.00000000
10	N	-0.84195694	3.86538979	0.00000000

3pyrimidine/SO2 (-5550.2)

1	C	1.02019464	4.14754583	-0.65430123
2	C	2.17720683	3.77103283	-1.33825588
3	H	0.23050183	4.72928569	-1.12849577
4	C	3.08178274	2.95728739	-0.65430123
5	H	2.35161637	4.07311903	-2.36734523
6	H	3.98043064	2.56426329	-1.12849577
7	N	0.80495605	3.78088846	0.62098861
8	C	2.17720683	-3.77103283	-1.33825588
9	C	1.02019464	-4.14754583	-0.65430123
10	H	2.35161637	-4.07311903	-2.36734523
11	H	0.23050183	-4.72928569	-1.12849577
12	S	-1.97603367	3.42259071	0.99290515
13	S	-1.97603367	-3.42259071	0.99290515
14	C	1.74840919	3.02833354	1.20469107
15	H	1.58058680	2.73765664	2.24129771
16	N	2.87186743	2.58755662	0.62098861
17	S	3.95206734	0.00000000	0.99290515
18	O	-2.50490669	-4.33862567	2.00979296
19	O	-2.18950543	-3.79233465	-0.41946900
20	O	-2.50490669	4.33862567	2.00979296
21	O	-2.18950543	3.79233465	-0.41946900
22	O	5.00981340	-0.00000000	2.00979296
23	O	4.37901086	0.00000000	-0.41946900
24	C	-4.10197738	-1.19025844	-0.65430123
25	C	-3.49681836	0.00000000	1.20469107
26	C	-4.35441365	-0.00000000	-1.33825588
27	H	-4.21093247	-2.16502241	-1.12849577
28	H	-3.16117360	0.00000000	2.24129771
29	C	-4.10197738	1.19025844	-0.65430123
30	H	-4.70323274	-0.00000000	-2.36734523
31	H	-4.21093247	2.16502241	-1.12849577
32	N	-3.67682348	-1.19333184	0.62098861
33	N	-3.67682348	1.19333184	0.62098861
34	C	3.08178274	-2.95728739	-0.65430123
35	H	3.98043064	-2.56426329	-1.12849577
36	C	1.74840919	-3.02833354	1.20469107
37	H	1.58058680	-2.73765664	2.24129771
38	N	2.87186743	-2.58755662	0.62098861
39	N	0.80495605	-3.78088846	0.62098861

4pyrimidine/SO2 (-7405.0)

1	C	1.19054082	5.50476715	-0.63916226
2	C	-0.00000000	5.83529942	-1.28919984
3	H	2.16789788	5.67329901	-1.09094148
4	C	-1.19054082	5.50476715	-0.63916226
5	H	-0.00000000	6.30510157	-2.26907942
6	H	-2.16789788	5.67329901	-1.09094148
7	N	1.19214586	4.93082458	0.57601829
8	S	-3.63460618	3.63460618	0.95432116
9	S	3.63460618	3.63460618	0.95432116
10	C	-5.50476715	-1.19054082	-0.63916226
11	C	-5.83529942	-0.00000000	-1.28919984
12	H	-5.67329901	-2.16789788	-1.09094148
13	C	-5.50476715	1.19054082	-0.63916226
14	H	-6.30510157	-0.00000000	-2.26907942
15	H	-5.67329901	2.16789788	-1.09094148

16	N	-4.93082458	-1.19214586	0.57601829
17	C	1.19054082	-5.50476715	-0.63916226
18	C	-1.19054082	-5.50476715	-0.63916226
19	C	0.00000000	-5.83529942	-1.28919984
20	H	2.16789788	-5.67329901	-1.09094148
21	H	-2.16789788	-5.67329901	-1.09094148
22	H	0.00000000	-6.30510157	-2.26907942
23	N	-1.19214586	-4.93082458	0.57601829
24	C	5.50476715	1.19054082	-0.63916226
25	C	5.83529942	0.00000000	-1.28919984
26	H	5.67329901	2.16789788	-1.09094148
27	C	5.50476715	-1.19054082	-0.63916226
28	H	6.30510157	0.00000000	-2.26907942
29	H	5.67329901	-2.16789788	-1.09094148
30	N	4.93082458	1.19214586	0.57601829
31	S	-3.63460618	-3.63460618	0.95432116
32	S	3.63460618	-3.63460618	0.95432116
33	C	-4.68162312	0.00000000	1.13591981
34	C	0.00000000	4.68162312	1.13591981
35	C	4.68162312	-0.00000000	1.13591981
36	C	-0.00000000	-4.68162312	1.13591981
37	H	-0.00000000	-4.22878897	2.12678194
38	H	4.22878897	-0.00000000	2.12678194
39	H	-4.22878897	0.00000000	2.12678194
40	H	0.00000000	4.22878897	2.12678194
41	N	-1.19214586	4.93082458	0.57601829
42	N	-4.93082458	1.19214586	0.57601829
43	N	1.19214586	-4.93082458	0.57601829
44	N	4.93082458	-1.19214586	0.57601829
45	O	-4.31473819	-4.31473819	2.06252074
46	O	-4.03030167	-4.03030167	-0.41309347
47	O	4.03030167	4.03030167	-0.41309347
48	O	4.31473819	4.31473819	2.06252074
49	O	4.31473819	-4.31473819	2.06252074
50	O	4.03030167	-4.03030167	-0.41309347
51	O	-4.31473819	4.31473819	2.06252074
52	O	-4.03030167	4.03030167	-0.41309347

5pyrimidine/SO2 (-9259.4)

1	C	1.80316995	5.54958646	0.99189934
2	C	1.02434903	7.00572176	-0.59653419
3	C	3.28914415	6.26984522	-0.59653419
4	C	2.29371877	7.05934051	-1.17553742
5	H	0.17258068	7.55201043	-1.00177851
6	H	4.29933966	6.21114516	-1.00177851
7	H	2.49299185	7.67263997	-2.05016721
8	C	-4.72076021	3.42983305	0.99189934
9	C	-6.34629607	3.13910091	-0.59653419
10	C	-4.94657571	5.06565070	-0.59653419
11	C	-6.00503371	4.36291237	-1.17553742
12	H	-7.12905838	2.49783355	-1.00177851
13	H	-4.57858106	6.00826441	-1.00177851
14	H	-6.52673739	4.74195228	-2.05016721
15	C	-4.72076021	-3.42983305	0.99189934
16	C	-4.94657571	-5.06565070	-0.59653419
17	C	-6.34629607	-3.13910091	-0.59653419
18	C	-6.00503371	-4.36291237	-1.17553742
19	H	-4.57858106	-6.00826441	-1.00177851
20	H	-7.12905838	-2.49783355	-1.00177851
21	H	-6.52673739	-4.74195228	-2.05016721

22	C	1.80316995	-5.54958646	0.99189934
23	C	3.28914415	-6.26984522	-0.59653419
24	C	1.02434903	-7.00572176	-0.59653419
25	C	2.29371877	-7.05934051	-1.17553742
26	H	4.29933966	-6.21114516	-1.00177851
27	H	0.17258068	-7.55201043	-1.00177851
28	H	2.49299185	-7.67263997	-2.05016721
29	C	5.83518053	0.00000000	0.99189934
30	C	6.97937859	1.19067326	-0.59653419
31	C	6.97937859	-1.19067326	-0.59653419
32	C	7.42262987	0.00000000	-1.17553742
33	H	7.23571910	2.16956559	-1.00177851
34	H	7.23571910	-2.16956559	-1.00177851
35	H	8.06749109	-0.00000000	-2.05016721
36	N	6.18724010	-1.19105166	0.48889933
37	N	6.18724010	1.19105166	0.48889933
38	N	3.04471978	5.51635982	0.48889933
39	N	0.77920489	6.25247022	0.48889933
40	N	-4.30549979	4.60034952	0.48889933
41	N	-5.70566500	2.67318745	0.48889933
42	N	-4.30549979	-4.60034952	0.48889933
43	N	-5.70566500	-2.67318745	0.48889933
44	N	0.77920489	-6.25247022	0.48889933
45	N	3.04471978	-5.51635982	0.48889933
46	H	-4.20926906	-3.05821299	1.87907530
47	H	-4.20926906	3.05821299	1.87907530
48	H	1.60779771	-4.94829255	1.87907530
49	H	5.20294268	0.00000000	1.87907530
50	H	1.60779771	4.94829255	1.87907530
51	S	-6.28809282	-0.00000000	0.86273397
52	S	-1.94312754	-5.98033165	0.86273397
53	S	5.08717395	-3.69604823	0.86273397
54	S	5.08717395	3.69604823	0.86273397
55	S	-1.94312754	5.98033165	0.86273397
56	O	-2.18243964	-6.71685857	2.10936150
57	O	-2.18127016	-6.71325925	-0.39853875
58	O	5.71063941	-4.14902239	-0.39853875
59	O	5.71370117	-4.15124690	2.10936150
60	O	-2.18243964	6.71685857	2.10936150
61	O	-2.18127016	6.71325925	-0.39853875
62	O	-7.05873850	-0.00000000	-0.39853875
63	O	-7.06252305	-0.00000000	2.10936150
64	O	5.71063941	4.14902239	-0.39853875
65	O	5.71370117	4.15124690	2.10936150

6pyrimidine/SO2 (-11113.5)

1	C	8.89448788	-0.00000000	-1.05901529
2	C	8.36901722	-1.19080376	-0.55417119
3	C	6.99314446	-0.00000000	0.83896943
4	C	8.36901722	1.19080376	-0.55417119
5	H	9.66679796	0.00000000	-1.82321517
6	H	8.68958791	-2.17049192	-0.90836409
7	H	6.23366270	-0.00000000	1.61950111
8	H	8.68958791	2.17049192	-0.90836409
9	C	3.49657223	-6.05624076	0.83896943
10	C	5.21577491	-6.65237964	-0.55417119
11	H	3.11683135	-5.39851026	1.61950111
12	C	4.44724394	-7.70285245	-1.05901529
13	H	6.22449510	-6.44015792	-0.90836409
14	C	3.15324230	-7.84318340	-0.55417119

15	H	4.83339898	-8.37169260	-1.82321517
16	H	2.46509282	-8.61064985	-0.90836409
17	C	-3.15324230	-7.84318340	-0.55417119
18	C	-3.49657223	-6.05624076	0.83896943
19	C	-4.44724394	-7.70285245	-1.05901529
20	H	-2.46509282	-8.61064985	-0.90836409
21	H	-3.11683135	-5.39851026	1.61950111
22	C	-5.21577491	-6.65237964	-0.55417119
23	H	-4.83339898	-8.37169260	-1.82321517
24	H	-6.22449510	-6.44015792	-0.90836409
25	C	-6.99314446	0.00000000	0.83896943
26	C	-8.36901722	-1.19080376	-0.55417119
27	H	-6.23366270	0.00000000	1.61950111
28	C	-8.89448788	0.00000000	-1.05901529
29	H	-8.68958791	-2.17049192	-0.90836409
30	C	-8.36901722	1.19080376	-0.55417119
31	H	-9.66679796	-0.00000000	-1.82321517
32	H	-8.68958791	2.17049192	-0.90836409
33	C	-5.21577491	6.65237964	-0.55417119
34	C	-3.49657223	6.05624076	0.83896943
35	C	-4.44724394	7.70285245	-1.05901529
36	H	-6.22449510	6.44015792	-0.90836409
37	H	-3.11683135	5.39851026	1.61950111
38	C	-3.15324230	7.84318340	-0.55417119
39	H	-4.83339898	8.37169260	-1.82321517
40	H	-2.46509282	8.61064985	-0.90836409
41	C	3.49657223	6.05624076	0.83896943
42	C	3.15324230	7.84318340	-0.55417119
43	H	3.11683135	5.39851026	1.61950111
44	C	4.44724394	7.70285245	-1.05901529
45	H	2.46509282	8.61064985	-0.90836409
46	C	5.21577491	6.65237964	-0.55417119
47	H	4.83339898	8.37169260	-1.82321517
48	H	6.22449510	6.44015792	-0.90836409
49	N	7.41938399	1.19069790	0.39602128
50	N	7.41938399	-1.19069790	0.39602128
51	N	4.74086663	-5.83002607	0.39602128
52	N	2.67851736	-7.02072397	0.39602128
53	N	-2.67851736	-7.02072397	0.39602128
54	N	-4.74086663	-5.83002607	0.39602128
55	N	-7.41938399	-1.19069790	0.39602128
56	N	-7.41938399	1.19069790	0.39602128
57	N	-4.74086663	5.83002607	0.39602128
58	N	-2.67851736	7.02072397	0.39602128
59	N	2.67851736	7.02072397	0.39602128
60	N	4.74086663	5.83002607	0.39602128
61	S	6.49284130	3.74864367	0.79890708
62	O	7.03861200	4.06374453	2.12468889
63	O	7.28024869	4.20325355	-0.36680805
64	O	7.03861200	-4.06374453	2.12468889
65	O	7.28024869	-4.20325355	-0.36680805
66	S	6.49284130	-3.74864367	0.79890708
67	S	0.00000000	7.49728735	0.79890708
68	S	-6.49284130	3.74864367	0.79890708
69	S	-6.49284130	-3.74864367	0.79890708
70	S	-0.00000000	-7.49728735	0.79890708
71	O	0.00000000	8.40650710	-0.36680805
72	O	-7.03861200	4.06374453	2.12468889
73	O	-7.28024869	4.20325355	-0.36680805
74	O	-7.28024869	-4.20325355	-0.36680805

75	O	-7.03861200	-4.06374453	2.12468889
76	O	-0.00000000	-8.40650710	-0.36680805
77	O	-0.00000000	-8.12748907	2.12468889
78	O	0.00000000	8.12748907	2.12468889

Pyrazine (-1464.6)

1	C	-1.13901242	0.69994857	-3.91159836
2	C	1.13901242	0.69994857	-3.91159836
3	C	-1.13901242	-0.69994857	-3.91159836
4	H	-2.07486760	1.25919750	-3.91162673
5	C	1.13901242	-0.69994857	-3.91159836
6	H	2.07486760	1.25919750	-3.91162673
7	H	-2.07486760	-1.25919750	-3.91162673
8	H	2.07486760	-1.25919750	-3.91162673
9	N	0.00000000	1.41328857	-3.91254916
10	N	-0.00000000	-1.41328857	-3.91254915

3pyrazine/SO2 (-5534.7)

1	C	-0.69467095	-3.92253448	1.14575850
2	C	-0.69467095	-3.92253448	-1.14575850
3	C	0.70462621	-3.91850371	1.14576450
4	H	-1.26515895	-3.91190482	2.07413835
5	C	0.70462621	-3.91850371	-1.14576450
6	H	-1.26515895	-3.91190482	-2.07413835
7	H	1.27505434	-3.90463307	2.07413648
8	H	1.27505434	-3.90463307	-2.07413648
9	C	2.90107416	2.55964840	1.14599826
10	C	2.90107416	2.55964840	-1.14599826
11	C	3.53613003	1.31272162	1.14629439
12	H	2.63233791	3.06269576	2.07449292
13	C	3.53613003	1.31272162	-1.14629439
14	H	2.63233791	3.06269576	-2.07449292
15	H	3.78690808	0.79898379	2.07397683
16	H	3.78690808	0.79898379	-2.07397683
17	C	-3.53886418	1.29877140	-1.14628227
18	C	-3.53886418	1.29877140	1.14628227
19	C	-2.90663167	2.54713979	-1.14598830
20	H	-3.78853451	0.78451205	-2.07397486
21	C	-2.90663167	2.54713979	1.14598830
22	H	-3.78853451	0.78451205	2.07397486
23	H	-2.63912741	3.05082182	-2.07449435
24	H	-2.63912741	3.05082182	2.07449435
25	N	2.57336869	3.17728912	0.00000000
26	N	3.84273452	0.68569064	0.00000000
27	N	-2.58031141	3.16554006	0.00000000
28	N	-3.84406060	0.67102940	0.00000000
29	N	-1.39393707	-3.91145006	0.00000000
30	N	1.40380841	-3.90339232	0.00000000
31	S	-0.00567380	4.30253214	0.00000000
32	S	-3.52616943	-2.08918696	0.00000000
33	S	3.53009990	-2.07440157	0.00000000
34	O	-0.00742325	5.05760082	-1.25994257
35	O	-0.00742325	5.05760082	1.25994257
36	O	4.20365461	-2.41724482	-1.25989166
37	O	4.20365461	-2.41724482	1.25989166
38	O	-4.19903902	-2.43334978	-1.25989177
39	O	-4.19903902	-2.43334978	1.25989177

4pyrazine/SO2 (-7383.8)

1	C	-0.69974603	5.13406105	1.14668019

2	C	-0.69974603	5.13406105	-1.14668019
3	C	0.69974603	5.13406105	1.14668019
4	H	-1.27305433	5.12861335	2.07327339
5	C	0.69974603	5.13406105	-1.14668019
6	H	-1.27305433	5.12861335	-2.07327339
7	H	1.27305433	5.12861335	2.07327339
8	H	1.27305433	5.12861335	-2.07327339
9	N	1.39615013	5.12373838	0.00000000
10	N	-1.39615013	5.12373838	0.00000000
11	C	-5.13406105	0.69974603	-1.14668019
12	C	-5.13406105	0.69974603	1.14668019
13	C	-5.13406105	-0.69974603	-1.14668019
14	H	-5.12861335	1.27305433	-2.07327339
15	C	-5.13406105	-0.69974603	1.14668019
16	H	-5.12861335	1.27305433	2.07327339
17	H	-5.12861335	-1.27305433	-2.07327339
18	H	-5.12861335	-1.27305433	2.07327339
19	N	-5.12373838	1.39615013	0.00000000
20	N	-5.12373838	-1.39615013	0.00000000
21	C	0.69974603	-5.13406105	-1.14668019
22	C	0.69974603	-5.13406105	1.14668019
23	C	-0.69974603	-5.13406105	-1.14668019
24	H	1.27305433	-5.12861335	-2.07327339
25	C	-0.69974603	-5.13406105	1.14668019
26	H	1.27305433	-5.12861335	2.07327339
27	H	-1.27305433	-5.12861335	-2.07327339
28	H	-1.27305433	-5.12861335	2.07327339
29	N	1.39615013	-5.12373838	0.00000000
30	N	-1.39615013	-5.12373838	0.00000000
31	C	5.13406105	0.69974603	1.14668019
32	C	5.13406105	0.69974603	-1.14668019
33	C	5.13406105	-0.69974603	1.14668019
34	H	5.12861335	1.27305433	2.07327339
35	C	5.13406105	-0.69974603	-1.14668019
36	H	5.12861335	1.27305433	-2.07327339
37	H	5.12861335	-1.27305433	2.07327339
38	H	5.12861335	-1.27305433	-2.07327339
39	N	5.12373838	1.39615013	0.00000000
40	N	5.12373838	-1.39615013	0.00000000
41	S	3.86790744	3.86790744	0.00000000
42	S	-3.86790744	3.86790744	0.00000000
43	S	3.86790744	-3.86790744	0.00000000
44	S	-3.86790744	-3.86790744	0.00000000
45	O	-4.40381842	4.40381842	-1.25945824
46	O	-4.40381842	-4.40381842	-1.25945824
47	O	4.40381842	4.40381842	-1.25945824
48	O	4.40381842	-4.40381842	-1.25945824
49	O	-4.40381842	-4.40381842	1.25945824
50	O	4.40381842	-4.40381842	1.25945824
51	O	4.40381842	4.40381842	1.25945824
52	O	-4.40381842	4.40381842	1.25945824

5pyrazine/SO2 (-9232.7)

1	C	-4.79334378	-4.34759291	-1.14766616
2	C	-4.79334378	-4.34759291	1.14766616
3	C	-5.61603126	-3.21526074	-1.14766616
4	H	-4.45376249	-4.81072546	-2.07343227
5	C	-5.61603126	-3.21526074	1.14766616
6	H	-4.45376249	-4.81072546	2.07343227
7	H	-5.95156009	-2.74918391	-2.07343227

8	H	-5.95156009	-2.74918391	2.07343227
9	C	2.65358189	-5.90222093	-1.14766616
10	C	2.65358189	-5.90222093	1.14766616
11	C	1.32244558	-6.33473333	-1.14766616
12	H	3.19898350	-5.72237576	-2.07343227
13	C	1.32244558	-6.33473333	1.14766616
14	H	3.19898350	-5.72237576	2.07343227
15	H	0.77549607	-6.50981456	-2.07343227
16	H	0.77549607	-6.50981456	2.07343227
17	C	6.43334758	0.69981977	-1.14766616
18	C	6.43334758	0.69981977	1.14766616
19	C	6.43334758	-0.69981977	-1.14766616
20	H	6.43084302	1.27410274	-2.07343227
21	C	6.43334758	-0.69981977	1.14766616
22	H	6.43084302	1.27410274	2.07343227
23	H	6.43084302	-1.27410274	-2.07343227
24	H	6.43084302	-1.27410274	2.07343227
25	C	1.32244558	6.33473333	1.14766616
26	C	1.32244558	6.33473333	-1.14766616
27	C	2.65358189	5.90222093	1.14766616
28	H	0.77549607	6.50981456	2.07343227
29	C	2.65358189	5.90222093	-1.14766616
30	H	0.77549607	6.50981456	-2.07343227
31	H	3.19898350	5.72237576	2.07343227
32	H	3.19898350	5.72237576	-2.07343227
33	C	-5.61603126	3.21526074	-1.14766616
34	C	-5.61603126	3.21526074	1.14766616
35	C	-4.79334378	4.34759291	-1.14766616
36	H	-5.95156009	2.74918391	-2.07343227
37	C	-4.79334378	4.34759291	1.14766616
38	H	-5.95156009	2.74918391	2.07343227
39	H	-4.45376249	4.81072546	-2.07343227
40	H	-4.45376249	4.81072546	2.07343227
41	O	-2.30956353	-7.10810564	1.25957451
42	O	-2.30956353	-7.10810564	-1.25957451
43	O	-7.47390456	-0.00000000	-1.25957451
44	O	-7.47390456	-0.00000000	1.25957451
45	O	-2.30956353	7.10810564	-1.25957451
46	O	-2.30956353	7.10810564	1.25957451
47	O	6.04651580	4.39305088	1.25957451
48	O	6.04651580	4.39305088	-1.25957451
49	O	6.04651580	-4.39305088	1.25957451
50	O	6.04651580	-4.39305088	-1.25957451
51	N	-6.01523569	-2.64789232	0.00000000
52	N	-4.37710530	-4.90258538	0.00000000
53	N	0.65948519	-6.53907283	0.00000000
54	N	3.31003585	-5.67785671	0.00000000
55	N	6.42281996	-1.39347694	0.00000000
56	N	6.42281996	1.39347694	0.00000000
57	N	3.31003585	5.67785671	0.00000000
58	N	0.65948519	6.53907283	0.00000000
59	N	-6.01523569	2.64789232	0.00000000
60	N	-4.37710530	4.90258538	0.00000000
61	S	5.43231410	-3.94680721	0.00000000
62	S	5.43231410	3.94680721	0.00000000
63	S	-2.07495935	6.38606822	0.00000000
64	S	-6.71470950	-0.00000000	0.00000000
65	S	-2.07495935	-6.38606822	0.00000000

6pyrazine/SO2 (-11081.2)

1	C	3.25794127	7.04258698	1.14821048
2	C	3.25794127	7.04258698	-1.14821048
3	C	4.47008859	6.34275339	1.14821048
4	H	2.75962300	7.33055119	2.07318372
5	C	4.47008859	6.34275339	-1.14821048
6	H	2.75962300	7.33055119	-2.07318372
7	H	4.96863206	6.05517922	2.07318372
8	H	4.96863206	6.05517922	-2.07318372
9	C	-4.47008859	6.34275339	-1.14821048
10	C	-4.47008859	6.34275339	1.14821048
11	C	-3.25794127	7.04258698	-1.14821048
12	H	-4.96863206	6.05517922	-2.07318372
13	C	-3.25794127	7.04258698	1.14821048
14	H	-4.96863206	6.05517922	2.07318372
15	H	-2.75962300	7.33055119	-2.07318372
16	H	-2.75962300	7.33055119	2.07318372
17	C	-7.72802986	-0.69983359	-1.14821048
18	C	-7.72802986	-0.69983359	1.14821048
19	C	-7.72802986	0.69983359	-1.14821048
20	H	-7.72825506	-1.27537198	-2.07318372
21	C	-7.72802986	0.69983359	1.14821048
22	H	-7.72825506	-1.27537198	2.07318372
23	H	-7.72825506	1.27537198	-2.07318372
24	H	-7.72825506	1.27537198	2.07318372
25	C	-3.25794127	-7.04258698	-1.14821048
26	C	-3.25794127	-7.04258698	1.14821048
27	C	-4.47008859	-6.34275339	-1.14821048
28	H	-2.75962300	-7.33055119	-2.07318372
29	C	-4.47008859	-6.34275339	1.14821048
30	H	-2.75962300	-7.33055119	2.07318372
31	H	-4.96863206	-6.05517922	-2.07318372
32	H	-4.96863206	-6.05517922	2.07318372
33	C	4.47008859	-6.34275339	-1.14821048
34	C	4.47008859	-6.34275339	1.14821048
35	C	3.25794127	-7.04258698	-1.14821048
36	H	4.96863206	-6.05517922	-2.07318372
37	C	3.25794127	-7.04258698	1.14821048
38	H	4.96863206	-6.05517922	2.07318372
39	H	2.75962300	-7.33055119	-2.07318372
40	H	2.75962300	-7.33055119	2.07318372
41	C	7.72802986	0.69983359	-1.14821048
42	C	7.72802986	0.69983359	1.14821048
43	C	7.72802986	-0.69983359	-1.14821048
44	H	7.72825506	1.27537198	-2.07318372
45	C	7.72802986	-0.69983359	1.14821048
46	H	7.72825506	1.27537198	2.07318372
47	H	7.72825506	-1.27537198	-2.07318372
48	H	7.72825506	-1.27537198	2.07318372
49	N	-7.71698122	1.39184419	0.00000000
50	N	-7.71698122	-1.39184419	0.00000000
51	N	-5.06386303	5.98717968	0.00000000
52	N	-2.65311818	7.37902387	0.00000000
53	N	2.65311818	7.37902387	0.00000000
54	N	5.06386303	5.98717968	0.00000000
55	N	7.71698122	1.39184419	0.00000000
56	N	7.71698122	-1.39184419	0.00000000
57	N	5.06386303	-5.98717968	0.00000000
58	N	2.65311818	-7.37902387	0.00000000
59	N	-2.65311818	-7.37902387	0.00000000
60	N	-5.06386303	-5.98717968	0.00000000

61	O	7.56918342	4.37007008	1.25975865
62	O	7.56918342	4.37007008	-1.25975865
63	O	0.00000000	8.74014017	-1.25975865
64	O	0.00000000	8.74014017	1.25975865
65	O	-7.56918342	4.37007008	-1.25975865
66	O	-7.56918342	4.37007008	1.25975865
67	O	-7.56918342	-4.37007008	1.25975865
68	O	-7.56918342	-4.37007008	-1.25975865
69	O	-0.00000000	-8.74014017	1.25975865
70	O	-0.00000000	-8.74014017	-1.25975865
71	O	7.56918342	-4.37007008	-1.25975865
72	O	7.56918342	-4.37007008	1.25975865
73	S	6.91112806	3.99014165	0.00000000
74	S	6.91112806	-3.99014165	0.00000000
75	S	-0.00000000	-7.98028329	0.00000000
76	S	-6.91112806	-3.99014165	0.00000000
77	S	-6.91112806	3.99014165	0.00000000
78	S	0.00000000	7.98028329	0.00000000

SO3 (-495.6)

1	O	1.06725512	-1.50542382	1.25751340
2	O	2.04412642	-3.45182255	0.00000000
3	O	1.06725512	-1.50542382	-1.25751340
4	S	1.39252909	-2.15412182	0.00000000

3pyridazine/SO3 (-5898.9)

1	C	4.54867614	0.86457335	0.00000000
2	C	4.54739360	-1.82341557	0.00000000
3	C	5.78856593	-1.17864057	0.00000000
4	C	5.78909400	0.20993352	0.00000000
5	H	4.45511222	1.94866752	0.00000000
6	H	4.41514997	-2.90184602	0.00000000
7	H	6.70765225	-1.75788716	0.00000000
8	H	6.71363467	0.78139048	0.00000000
9	O	-3.99965142	-0.00362413	0.00000000
10	O	-1.84933756	-0.12985029	-1.26259986
11	N	3.41389396	-1.11418868	0.00000000
12	N	3.38214811	0.20991897	0.00000000
13	O	0.81221513	1.66649845	1.26259986
14	C	-0.69457259	4.84986617	0.00000000
15	C	-3.02308055	3.50698241	0.00000000
16	C	-1.87355029	5.60236543	0.00000000
17	H	0.30549739	5.27455504	0.00000000
18	C	-3.07635476	4.90853571	0.00000000
19	H	-3.91515170	2.88390660	0.00000000
20	H	-1.83145119	6.68794082	0.00000000
21	H	-4.03352134	5.42348293	0.00000000
22	N	-0.74203129	3.51361324	0.00000000
23	N	-1.87286922	2.82406670	0.00000000
24	S	1.49395660	-2.08150802	0.00000000
25	O	1.03712243	-1.53664816	1.26259986
26	O	2.00296429	-3.46198768	0.00000000
27	S	1.05566052	2.33455839	0.00000000
28	S	-2.54961713	-0.25305036	0.00000000
29	C	-3.85282101	-3.02645059	0.00000000
30	C	-3.91501564	-4.42372486	0.00000000
31	H	-4.72064735	-2.37270902	0.00000000
32	C	-1.52559558	-4.37155576	0.00000000
33	C	-2.71273923	-5.11846923	0.00000000
34	H	-4.87620106	-4.93005367	0.00000000

35	H	-0.53996053	-4.83257412	0.00000000
36	H	-2.68011333	-6.20487341	0.00000000
37	N	-2.67186267	-2.39942456	0.00000000
38	N	-1.50927889	-3.03398566	0.00000000
39	O	1.99668713	3.46561179	0.00000000
40	O	1.03712243	-1.53664816	-1.26259986
41	O	-1.84933756	-0.12985029	1.26259986
42	O	0.81221513	1.66649845	-1.26259986

4pyridazine/SO3 (-7870.9)

1	C	-6.24972544	6.39780335	0.00000000
2	C	-6.85590401	7.66285384	0.00000000
3	H	-6.81530654	5.46887009	0.00000000
4	C	-4.65257171	8.56202405	0.00000000
5	C	-6.03291926	8.78035072	0.00000000
6	H	-7.93921311	7.75069419	0.00000000
7	H	-3.90362299	9.34813564	0.00000000
8	H	-6.42744358	9.79258639	0.00000000
9	C	1.41358797	8.20522987	0.00000000
10	C	2.68388694	8.80029702	0.00000000
11	H	0.48831201	8.77687577	0.00000000
12	C	3.56437029	6.58893236	0.00000000
13	C	3.79420561	7.96743005	0.00000000
14	H	2.78154911	9.88276137	0.00000000
15	H	4.34388367	5.83310474	0.00000000
16	H	4.80976215	8.35329258	0.00000000
17	C	3.07446591	0.50345856	0.00000000
18	C	1.49604745	-1.67465897	0.00000000
19	C	3.69155466	-0.75628823	0.00000000
20	H	3.63256669	1.43712049	0.00000000
21	C	2.87821150	-1.88089117	0.00000000
22	H	0.75387454	-2.46714953	0.00000000
23	H	4.77558353	-0.83478570	0.00000000
24	H	3.28158749	-2.88963568	0.00000000
25	C	-4.56983452	-1.31082954	0.00000000
26	C	-5.84533405	-1.89434434	0.00000000
27	H	-3.65028460	-1.89153297	0.00000000
28	C	-6.70497529	0.32495960	0.00000000
29	C	-6.94784896	-1.05126467	0.00000000
30	H	-5.95274945	-2.97588804	0.00000000
31	H	-7.47759106	1.08780719	0.00000000
32	H	-7.96697977	-1.42761307	0.00000000
33	N	-5.45029953	0.79023899	0.00000000
34	N	-4.37720041	0.01494692	0.00000000
35	N	0.99783108	-0.43271424	0.00000000
36	N	1.74393577	0.66111690	0.00000000
37	N	2.31385707	6.11279920	0.00000000
38	N	1.23362113	6.87785916	0.00000000
39	N	-4.16510999	7.31593086	0.00000000
40	N	-4.92071387	6.22852907	0.00000000
41	O	-1.98248862	6.29466047	1.26102761
42	O	-1.98248862	6.29466047	-1.26102761
43	O	-4.39144107	2.95752646	-1.26098768
44	O	-4.39144107	2.95752646	1.26098768
45	O	-1.19250089	0.57045892	1.26110782
46	O	-1.19250089	0.57045892	-1.26110782
47	O	1.26607455	3.94542861	-1.26086606
48	O	1.26607455	3.94542861	1.26086606
49	S	-2.07612503	7.00383385	0.00000000
50	S	-5.10200430	2.87506519	0.00000000

51	S	-1.09265599	-0.13773230	0.00000000
52	S	1.97650439	4.03216864	0.00000000
53	O	-1.43589424	-1.57152149	0.00000000
54	O	3.40212414	3.65614532	0.00000000
55	O	-1.72154235	8.43485962	0.00000000
56	O	-6.52714463	3.25249375	0.00000000

5pyridazine/SO3 (-9834.4)

1	C	-11.43618661	-0.15716880	0.00000000
2	C	-11.00401999	-2.80943766	0.00000000
3	C	-12.55566590	-0.98889513	0.00000000
4	H	-11.48567901	0.92743409	0.00000000
5	C	-12.33025441	-2.36025189	0.00000000
6	H	-10.74232533	-3.86298545	0.00000000
7	H	-13.55572175	-0.56472071	0.00000000
8	H	-13.15032316	-3.07341050	0.00000000
9	C	-5.35476993	5.44403596	0.00000000
10	C	-6.50535577	6.23206438	0.00000000
11	H	-4.34505946	5.84308928	0.00000000
12	C	-7.72252793	4.17265351	0.00000000
13	C	-7.72894119	5.57290256	0.00000000
14	H	-6.42936716	7.31567834	0.00000000
15	H	-8.63331895	3.58185338	0.00000000
16	H	-8.66994142	6.11665379	0.00000000
17	C	1.84285689	1.32527731	0.00000000
18	C	2.26163656	2.65564883	0.00000000
19	H	2.51874922	0.47558865	0.00000000
20	C	-0.06284261	3.22060707	0.00000000
21	C	1.27488138	3.63448362	0.00000000
22	H	3.32041502	2.89860651	0.00000000
23	H	-0.89332308	3.91984251	0.00000000
24	H	1.52120246	4.69301366	0.00000000
25	C	0.18804144	-6.79338365	0.00000000
26	C	1.58170002	-6.74061767	0.00000000
27	H	-0.38471081	-7.71576883	0.00000000
28	C	1.33173034	-4.36141525	0.00000000
29	C	2.17125373	-5.48215465	0.00000000
30	H	2.16647054	-7.65607912	0.00000000
31	H	1.71203951	-3.34470413	0.00000000
32	H	3.25091280	-5.35781066	0.00000000
33	C	-8.07178343	-7.74174032	0.00000000
34	C	-5.41271779	-8.13031793	0.00000000
35	C	-7.61673235	-9.06010958	0.00000000
36	H	-9.12073312	-7.46144947	0.00000000
37	C	-6.24136316	-9.25916944	0.00000000
38	H	-4.32929815	-8.19942921	0.00000000
39	H	-8.32309239	-9.88535379	0.00000000
40	H	-5.80891232	-10.25621527	0.00000000
41	N	0.53717502	1.02510571	0.00000000
42	N	-0.42518273	1.92832451	0.00000000
43	N	-5.44784034	4.10744020	0.00000000
44	N	-6.59210242	3.44914262	0.00000000
45	N	-10.20224934	-0.67906546	0.00000000
46	N	-9.95230602	-1.97531363	0.00000000
47	N	-7.20165648	-6.72308715	0.00000000
48	N	-5.89010496	-6.87584471	0.00000000
49	N	-0.53397366	-5.66483729	0.00000000
50	N	-0.00738846	-4.45400907	0.00000000
51	S	-2.64183937	-5.86123763	0.00000000
52	S	-8.00192151	-4.76314641	0.00000000

53	S	-8.57342765	0.67851428	0.00000000
54	S	-3.64455745	3.00921344	0.00000000
55	S	0.00444211	-1.02189898	0.00000000
56	O	-8.77143183	-4.90294141	1.23951360
57	O	-8.77143183	-4.90294141	-1.23951360
58	O	-8.94183304	1.36763098	-1.23984427
59	O	-8.94183304	1.36763098	1.23984427
60	O	-3.10852639	3.57885773	-1.23958618
61	O	-3.10852639	3.57885773	1.23958618
62	O	0.70429614	-1.37076334	1.23956245
63	O	0.70429614	-1.37076334	-1.23956245
64	O	-2.73214449	-6.63799569	1.23962255
65	O	-2.73214449	-6.63799569	-1.23962255
66	O	-4.19874178	1.67634558	0.00000000
67	O	-1.43139327	-0.87338900	0.00000000
68	O	-2.99167120	-4.46073476	0.00000000
69	O	-6.76325105	-4.02198135	0.00000000
70	O	-7.49142444	-0.27694768	0.00000000

6pyridazine/SO3 (-11802.5)

1	C	-5.66028534	4.20228319	0.00000000
2	C	-3.04173895	3.58601596	0.00000000
3	C	-4.75207772	5.26066798	0.00000000
4	H	-6.73890445	4.32531009	0.00000000
5	C	-3.39999702	4.94051750	0.00000000
6	H	-2.00647038	3.25854749	0.00000000
7	H	-5.10636051	6.28748529	0.00000000
8	H	-2.63145452	5.70879538	0.00000000
9	C	-12.28780327	-1.23536801	0.00000000
10	C	-12.65845128	0.10931214	0.00000000
11	H	-12.99236151	-2.06137224	0.00000000
12	C	-10.31404296	0.59270897	0.00000000
13	C	-11.63780245	1.05210364	0.00000000
14	H	-13.70808184	0.38895541	0.00000000
15	H	-9.46577812	1.27062514	0.00000000
16	H	-11.84553917	2.11873280	0.00000000
17	C	-10.90113583	-9.66898352	0.00000000
18	C	-12.27422748	-9.42504314	0.00000000
19	H	-10.45906305	-10.66054669	0.00000000
20	C	-11.70159919	-7.10116762	0.00000000
21	C	-12.68603875	-8.09789750	0.00000000
22	H	-12.97844882	-10.25203813	0.00000000
23	H	-11.94531495	-6.04319213	0.00000000
24	H	-13.73835024	-7.82670436	0.00000000
25	C	-2.87219667	-12.46548972	0.00000000
26	C	-3.78045871	-13.52382736	0.00000000
27	H	-1.79358402	-12.58857066	0.00000000
28	C	-5.49071887	-11.84909209	0.00000000
29	C	-5.13252458	-13.20360992	0.00000000
30	H	-3.42622967	-14.55066323	0.00000000
31	H	-6.52596388	-11.52156271	0.00000000
32	H	-5.90110230	-13.97185300	0.00000000
33	C	3.75546509	-7.02796381	0.00000000
34	C	4.12614466	-8.37263401	0.00000000
35	H	4.46000101	-6.20194079	0.00000000
36	C	1.78174696	-8.85609354	0.00000000
37	C	3.10551817	-9.31545251	0.00000000
38	H	5.17578180	-8.65225257	0.00000000
39	H	0.93349548	-9.53402675	0.00000000
40	H	3.31328365	-10.38207616	0.00000000

41	C	2.36868165	1.40560808	0.00000000
42	C	3.16897991	-1.16219985	0.00000000
43	C	3.74177652	1.16158607	0.00000000
44	H	1.92667214	2.39719978	0.00000000
45	C	4.15352314	-0.16558594	0.00000000
46	H	3.41264913	-2.22018539	0.00000000
47	H	4.44604408	1.98854204	0.00000000
48	H	5.20581317	-0.43686274	0.00000000
49	O	-1.20108386	-10.08237526	1.23900838
50	O	-1.20108386	-10.08237526	-1.23900838
51	O	2.70068434	-4.30752192	1.23914562
52	O	2.70068434	-4.30752192	-1.23914562
53	O	-0.53698592	1.63366213	1.23901794
54	O	-0.53698592	1.63366213	-1.23901794
55	O	-7.33127644	1.81907123	1.23901178
56	O	-7.33127644	1.81907123	-1.23901178
57	O	-11.23311577	-3.95584080	-1.23916417
58	O	-11.23311577	-3.95584080	1.23916417
59	O	-7.99550986	-9.89690173	1.23901826
60	O	-7.99550986	-9.89690173	-1.23901826
61	S	-7.99740554	-9.11336115	0.00000000
62	S	-1.87778589	-9.68740367	0.00000000
63	S	1.99689053	-4.65195667	0.00000000
64	S	-0.53511482	0.85012122	0.00000000
65	S	-6.65455125	1.42413717	0.00000000
66	S	-10.52930882	-3.61143139	0.00000000
67	N	-3.31133891	-11.19982404	0.00000000
68	N	-4.58653478	-10.85723536	0.00000000
69	N	-10.03397877	-8.64776192	0.00000000
70	N	-10.38770443	-7.37568877	0.00000000
71	N	-10.99282278	-1.57779854	0.00000000
72	N	-9.99641440	-0.71124104	0.00000000
73	N	-5.22108228	2.93664066	0.00000000
74	N	-3.94586864	2.59410930	0.00000000
75	N	1.50144372	0.38444129	0.00000000
76	N	1.85511695	-0.88763777	0.00000000
77	N	2.46047409	-6.68556977	0.00000000
78	N	1.46408957	-7.55215103	0.00000000
79	O	-9.08737189	-3.51811706	0.00000000
80	O	-7.45442795	-7.77441332	0.00000000
81	O	-2.75259216	-8.53707666	0.00000000
82	O	0.55495756	-4.74533369	0.00000000
83	O	-1.07813477	-0.48880958	0.00000000
84	O	-5.77968083	0.27385918	0.00000000

3pyrimidine/SO3 (-5968.8)

1	N	-3.93393602	-1.44363609	0.31177751
2	C	-4.87222541	1.08132542	-0.29277779
3	C	-5.74004918	-0.00224869	-0.36075694
4	C	-5.21599411	-1.26014235	-0.04681762
5	O	-1.09459743	-2.26932797	-0.97389038
6	H	-5.14957638	2.10913979	-0.50897847
7	H	-6.77925529	0.13039434	-0.64639601
8	H	-5.83886290	-2.15322796	-0.08150681
9	O	4.68842235	1.05576706	-0.08688485
10	O	2.51259439	0.18671480	-0.97389038
11	N	-3.59222076	0.88023734	0.06438572
12	C	-3.15676939	-0.36196128	0.35555005
13	O	-1.06551982	-2.66879506	1.48730376
14	C	1.49965741	-4.76013369	-0.29277779

15	N	3.21719353	-2.68507049	0.31177751
16	C	2.87197202	-4.96990407	-0.36075694
17	H	0.74821956	-5.51423385	-0.50897847
18	C	3.69931234	-3.88711223	-0.04681762
19	O	2.84400424	0.41163030	1.48730376
20	H	3.27670283	-5.93620447	-0.64639601
21	H	4.78418157	-3.97998962	-0.08150681
22	N	1.03380248	-3.55107309	0.06438572
23	C	1.89185236	-2.55286185	0.35555005
24	S	-2.27948423	2.47434453	0.13512680
25	O	-1.77848441	2.25716477	1.48730376
26	O	-3.25853227	3.53240933	-0.08688485
27	S	-1.00310310	-3.21126352	0.13512680
28	S	3.28258734	0.73691899	0.13512680
29	C	3.37256799	3.67880827	-0.29277779
30	C	2.86807717	4.97215276	-0.36075694
31	H	4.40135682	3.40509407	-0.50897847
32	N	0.71674248	4.12870657	0.31177751
33	C	1.51668177	5.14725458	-0.04681762
34	H	3.50255246	5.80581013	-0.64639601
35	O	-1.41799696	2.08261317	-0.97389038
36	H	1.05468133	6.13321758	-0.08150681
37	N	2.55841828	2.67083575	0.06438572
38	C	1.26491703	2.91482313	0.35555005
39	O	-1.42989009	-4.58817640	-0.08688485
40	H	0.65039190	2.06799579	0.64386504
41	H	1.46574094	-1.59725381	0.64386504
42	H	-2.11613284	-0.47074198	0.64386504

4pyrimidine/SO3 (-7973.7)

1	N	-3.37852444	-3.96273944	1.57909800
2	C	-3.09101158	-4.78389057	0.55035695
3	O	5.21985098	0.23443446	0.01878420
4	C	-3.11262067	-2.96681435	-0.98756927
5	C	-2.94486340	-4.32696919	-0.76075314
6	H	-2.95979906	-5.83626361	0.79329837
7	H	-3.00680709	-2.48402725	-1.95358250
8	H	-2.67865271	-4.99564738	-1.57220986
9	N	-3.96273944	3.37852444	1.57909800
10	C	-4.78389057	3.09101158	0.55035695
11	O	-5.21985098	-0.23443446	0.01878420
12	C	-2.96681435	3.11262067	-0.98756927
13	C	-4.32696919	2.94486340	-0.76075314
14	H	-5.83626361	2.95979906	0.79329837
15	H	-2.48402725	3.00680709	-1.95358250
16	H	-4.99564738	2.67865271	-1.57220986
17	N	3.37852444	3.96273944	1.57909800
18	C	3.11262067	2.96681435	-0.98756927
19	C	3.09101158	4.78389057	0.55035695
20	O	0.23443446	-5.21985098	0.01878420
21	C	2.94486340	4.32696919	-0.76075314
22	H	3.00680709	2.48402725	-1.95358250
23	H	2.95979906	5.83626361	0.79329837
24	H	2.67865271	4.99564738	-1.57220986
25	N	3.96273944	-3.37852444	1.57909800
26	C	4.78389057	-3.09101158	0.55035695
27	O	-0.23443446	5.21985098	0.01878420
28	C	2.96681435	-3.11262067	-0.98756927
29	C	4.32696919	-2.94486340	-0.76075314
30	H	5.83626361	-2.95979906	0.79329837

31	H	2.48402725	-3.00680709	-1.95358250
32	H	4.99564738	-2.67865271	-1.57220986
33	N	2.16522174	-3.40819566	0.04946629
34	C	2.67347668	-3.51722659	1.29542847
35	N	3.40819566	2.16522174	0.04946629
36	C	3.51722659	2.67347668	1.29542847
37	N	-2.16522174	3.40819566	0.04946629
38	C	-2.67347668	3.51722659	1.29542847
39	N	-3.40819566	-2.16522174	0.04946629
40	C	-3.51722659	-2.67347668	1.29542847
41	O	-2.91964841	0.34326384	0.78142938
42	O	-3.35719905	-0.11725253	-1.63843043
43	O	0.11725253	-3.35719905	-1.63843043
44	O	-0.34326384	-2.91964841	0.78142938
45	O	2.91964841	-0.34326384	0.78142938
46	O	3.35719905	0.11725253	-1.63843043
47	O	-0.11725253	3.35719905	-1.63843043
48	O	0.34326384	2.91964841	0.78142938
49	S	-3.78208236	-0.22947905	-0.24498785
50	S	0.22947905	-3.78208236	-0.24498785
51	S	3.78208236	0.22947905	-0.24498785
52	S	-0.22947905	3.78208236	-0.24498785
53	H	-3.73403043	-1.96428289	2.08967138
54	H	1.96428289	-3.73403043	2.08967138
55	H	3.73403043	1.96428289	2.08967138
56	H	-1.96428289	3.73403043	2.08967138

5pyrimidine/SO3 (-9965.1)

1	C	2.13193925	-4.98396464	-0.99009441
2	N	3.04584778	-5.50795129	1.56712413
3	C	3.48432226	-5.21721006	-0.78339371
4	H	1.68256267	-4.76095928	-1.95259533
5	C	3.89881942	-5.48331295	0.52466386
6	O	3.40336235	-2.18963158	0.78270498
7	H	4.18830856	-5.16599057	-1.60682124
8	H	4.94669168	-5.66474660	0.75095529
9	C	-4.08122659	-3.56772449	-0.99009441
10	C	-3.88514683	-4.92599395	-0.78339371
11	H	-4.00800089	-3.07142952	-1.95259533
12	N	-4.29715424	-4.59882394	1.56712413
13	C	-4.01013905	-5.40243451	0.52466386
14	H	-3.61889046	-5.57969703	-1.60682124
15	O	-1.03076657	-3.91342331	0.78270498
16	H	-3.85888238	-6.45508633	0.75095529
17	C	-4.65427599	2.77898964	-0.99009441
18	C	-5.88547504	2.17277837	-0.78339371
19	H	-4.15964345	2.86271144	-1.95259533
20	N	-5.70163516	2.66572179	1.56712413
21	C	-6.37722166	2.14442480	0.52466386
22	H	-6.42490587	1.71754815	-1.60682124
23	O	-4.04041113	-0.22899704	0.78270498
24	H	-7.33161214	1.67528385	0.75095529
25	C	1.20472583	5.28523454	-0.99009441
26	C	0.24772321	6.26884483	-0.78339371
27	H	1.43719986	4.84068249	-1.95259533
28	N	0.77334991	6.24633061	1.56712413
29	C	0.06879931	6.72776192	0.52466386
30	H	-0.35191974	6.64120017	-1.60682124
31	O	-1.46634484	3.77189535	0.78270498
32	H	-0.67230313	7.49046869	0.75095529

33	C	5.39883751	0.48746495	-0.99009441
34	N	6.17959169	1.19472283	1.56712413
35	C	6.03857642	1.70158081	-0.78339371
36	H	5.04788181	0.12899486	-1.95259533
37	C	6.41974197	2.01356074	0.52466386
38	O	3.13416018	2.56015658	0.78270498
39	H	6.20740751	2.38693928	-1.60682124
40	H	6.91610596	2.95408039	0.75095529
41	N	-3.99932370	3.30439605	0.06080591
42	C	-4.52583785	3.22280274	1.30166257
43	N	-4.37852639	-2.78246832	0.06080591
44	C	-4.46362835	-3.30842677	1.30166257
45	N	1.29324557	-5.02405605	0.06080591
46	C	1.76716382	-5.26752293	1.30166257
47	N	5.17779610	-0.32256908	0.06080591
48	C	5.55579566	0.05291856	1.30166257
49	N	1.90680841	4.82469739	0.06080591
50	C	1.66650673	5.30022839	1.30166257
51	S	3.43983653	3.57322269	-0.21920196
52	S	4.46130467	-2.16729241	-0.21920196
53	S	-0.68259861	-4.91268307	-0.21920196
54	S	-4.88317382	-0.86891270	-0.21920196
55	S	-2.33536878	4.37566548	-0.21920196
56	O	5.68218737	-2.91580036	0.08135717
57	O	4.07630372	-2.05381162	-1.62364128
58	O	-0.69364381	-4.51145791	-1.62364128
59	O	-1.01719846	-6.30511319	0.08135717
60	O	-4.50499916	-0.73442270	-1.62364128
61	O	-6.31085060	-0.98097389	0.08135717
62	O	-2.88312170	5.69883798	0.08135717
63	O	-2.09059880	4.05755972	-1.62364128
64	O	4.52898339	4.50304947	0.08135717
65	O	3.21293806	3.24213251	-1.62364128
66	H	-4.68913146	-2.61350617	2.10647402
67	H	-3.93461339	3.65201121	2.10647402
68	H	2.25740665	4.87057322	2.10647402
69	H	5.32976742	-0.64183140	2.10647402
70	H	1.03657077	-5.26724685	2.10647402

6pyrimidine/SO3 (-11955.7)

1	C	-5.92536132	3.19252686	-0.96601103
2	N	-6.54397261	4.24602721	1.51637645
3	C	-5.87438059	4.56631996	-0.77509126
4	H	-5.69038095	2.68855863	-1.89843787
5	C	-6.19170015	5.05356752	0.49604935
6	O	-2.82460638	-4.99976135	0.94953896
7	H	-5.57125179	5.22930290	-1.57793307
8	H	-6.14514894	6.11861448	0.70984659
9	C	-5.72749002	-3.53525000	-0.96601103
10	C	-6.89173938	-2.80420285	-0.77509126
11	H	-5.17355055	-3.58373514	-1.89843787
12	N	-6.94915373	-3.54423292	1.51637645
13	C	-7.47236793	-2.83538586	0.49604935
14	H	-7.31433505	-2.21019412	-1.57793307
15	O	2.91761715	-4.94606155	0.94953896
16	H	-8.37145005	-2.26254784	0.70984659
17	C	0.19787129	-6.72777685	-0.96601103
18	C	-1.01735879	-7.37052280	-0.77509126
19	H	0.51683040	-6.27229378	-1.89843787
20	N	-0.40518111	-7.79026013	1.51637645

21	C	-1.28066778	-7.88895338	0.49604935
22	H	-1.74308326	-7.43949702	-1.57793307
23	O	5.74222353	0.05369979	0.94953896
24	H	-2.22630111	-8.38116233	0.70984659
25	C	5.92536132	-3.19252686	-0.96601103
26	C	5.87438059	-4.56631996	-0.77509126
27	H	5.69038095	-2.68855863	-1.89843787
28	N	6.54397261	-4.24602721	1.51637645
29	C	6.19170015	-5.05356752	0.49604935
30	H	5.57125179	-5.22930290	-1.57793307
31	O	2.82460638	4.99976135	0.94953896
32	H	6.14514894	-6.11861448	0.70984659
33	C	5.72749002	3.53525000	-0.96601103
34	C	6.89173938	2.80420285	-0.77509126
35	H	5.17355055	3.58373514	-1.89843787
36	N	6.94915373	3.54423292	1.51637645
37	C	7.47236793	2.83538586	0.49604935
38	H	7.31433505	2.21019412	-1.57793307
39	O	-2.91761715	4.94606155	0.94953896
40	H	8.37145005	2.26254784	0.70984659
41	C	-0.19787129	6.72777685	-0.96601103
42	N	0.40518111	7.79026013	1.51637645
43	C	1.01735879	7.37052280	-0.77509126
44	H	-0.51683040	6.27229378	-1.89843787
45	C	1.28066778	7.88895338	0.49604935
46	O	-5.74222353	-0.05369979	0.94953896
47	H	1.74308326	7.43949702	-1.57793307
48	H	2.22630111	8.38116233	0.70984659
49	O	7.95737652	-0.34916857	-0.11946078
50	O	5.89467841	-0.30865953	-1.52375969
51	O	4.28107712	6.71670593	-0.11946078
52	O	3.21464619	4.95061149	-1.52375969
53	O	-3.67629941	7.06587450	-0.11946078
54	O	-2.68003222	5.25927101	-1.52375969
55	O	-7.95737652	0.34916857	-0.11946078
56	O	-5.89467841	0.30865953	-1.52375969
57	O	-3.21464619	-4.95061149	-1.52375969
58	O	-4.28107712	-6.71670593	-0.11946078
59	O	3.67629941	-7.06587450	-0.11946078
60	O	2.68003222	-5.25927101	-1.52375969
61	S	2.87749836	-5.84306192	-0.19895642
62	S	6.49898924	-0.42954428	-0.19895642
63	S	3.62149088	5.41351763	-0.19895642
64	S	-2.87749836	5.84306192	-0.19895642
65	S	-6.49898924	0.42954428	-0.19895642
66	S	-3.62149088	-5.41351763	-0.19895642
67	N	6.28134559	-2.40218706	0.06365669
68	C	6.57337145	-2.94193623	1.26703408
69	N	1.06031778	-6.64089839	0.06365669
70	C	0.73889422	-7.16367478	1.26703408
71	N	-5.22102782	-4.23871132	0.06365669
72	C	-5.83447724	-4.22173856	1.26703408
73	N	-6.28134559	2.40218706	0.06365669
74	C	-6.57337145	2.94193623	1.26703408
75	N	-1.06031778	6.64089839	0.06365669
76	C	-0.73889422	7.16367478	1.26703408
77	N	5.22102782	4.23871132	0.06365669
78	C	5.83447724	4.22173856	1.26703408
79	H	-6.84314403	2.24578475	2.05714798
80	H	-1.47666537	7.04922894	2.05714798

81	H	5.36647865	4.80344420	2.05714798
82	H	6.84314403	-2.24578475	2.05714798
83	H	1.47666537	-7.04922894	2.05714798
84	H	-5.36647865	-4.80344420	2.05714798

3pyrazine/SO3 (-5967.2)

1	C	-4.11765915	-1.11661771	0.95449361
2	C	-3.49257655	0.42756222	-1.16484037
3	C	-4.35043737	-0.66351467	-1.27452906
4	N	-4.67938943	-1.42829718	-0.21987782
5	O	-1.41287601	-1.91151620	-0.89321308
6	H	-3.20923357	1.06345527	-1.99705656
7	H	-4.78332447	-0.92536262	-2.23760508
8	H	1.59243625	2.27722232	2.03939237
9	O	4.38033444	0.85839454	0.04677165
10	O	2.36185959	-0.26782841	-0.89321308
11	N	-2.97388488	0.72672451	0.03817203
12	C	-3.24847561	-0.03141958	1.10629933
13	O	-1.20061632	-2.25544528	1.57169993
14	C	1.37600853	-3.23844113	-1.16484037
15	C	3.02584888	-3.00768858	0.95449361
16	C	2.74983924	-3.43583195	-1.27452906
17	H	0.68363750	-3.31100542	-1.99705656
18	N	3.57663636	-3.33832153	-0.21987782
19	O	2.55358107	0.08795841	1.57169993
20	H	3.19304977	-3.67979919	-2.23760508
21	H	-2.76835050	0.24047908	2.03939237
22	N	0.85758055	-2.93882211	0.03817203
23	C	1.65144797	-2.79755261	1.10629933
24	S	-1.87000942	2.38427724	0.22138099
25	O	-1.35296475	2.16748688	1.57169993
26	O	-2.93355869	3.36428363	0.04677165
27	S	-1.12983995	-2.81161428	0.22138099
28	S	2.99984936	0.42733704	0.22138099
29	C	2.11656802	2.81087891	-1.16484037
30	C	1.60059813	4.09934662	-1.27452906
31	H	2.52559607	2.24755015	-1.99705656
32	C	1.09181028	4.12430628	0.95449361
33	N	1.10275308	4.76661871	-0.21987782
34	H	1.59027470	4.60516181	-2.23760508
35	O	-0.94898358	2.17934461	-0.89321308
36	H	1.17591426	-2.51770140	2.03939237
37	N	2.11630433	2.21209759	0.03817203
38	C	1.59702765	2.82897220	1.10629933
39	O	-1.44677574	-4.22267817	0.04677165
40	H	3.68901993	-2.89468061	1.80891208
41	H	0.66235699	4.64212529	1.80891208
42	H	-4.35137692	-1.74744467	1.80891208

4pyrazine/SO3 (-7952.0)

1	C	-2.18375029	-4.53019325	-0.47339364
2	N	-3.08253964	-5.19299450	-1.22060607
3	O	5.50273387	0.90628336	0.47164164
4	C	-4.69572122	-3.53606307	-0.58309787
5	C	-4.32800072	-4.69268511	-1.27002329
6	H	-2.81441513	1.79418100	0.82738336
7	H	-5.68960884	-3.09977275	-0.60400144
8	H	-5.05969626	-5.22560854	-1.87488871
9	C	-4.53019325	2.18375029	-0.47339364

10	N	-5.19299450	3.08253964	-1.22060607
11	O	-5.50273387	-0.90628336	0.47164164
12	C	-3.53606307	4.69572122	-0.58309787
13	C	-4.69268511	4.32800072	-1.27002329
14	H	-1.79418100	-2.81441513	0.82738336
15	H	-3.09977275	5.68960884	-0.60400144
16	H	-5.22560854	5.05969626	-1.87488871
17	C	2.18375029	4.53019325	-0.47339364
18	C	4.69572122	3.53606307	-0.58309787
19	N	3.08253964	5.19299450	-1.22060607
20	O	0.90628336	-5.50273387	0.47164164
21	C	4.32800072	4.69268511	-1.27002329
22	H	5.68960884	3.09977275	-0.60400144
23	H	2.81441513	-1.79418100	0.82738336
24	H	5.05969626	5.22560854	-1.87488871
25	C	4.53019325	-2.18375029	-0.47339364
26	N	5.19299450	-3.08253964	-1.22060607
27	O	-0.90628336	5.50273387	0.47164164
28	C	3.53606307	-4.69572122	-0.58309787
29	C	4.69268511	-4.32800072	-1.27002329
30	H	1.79418100	2.81441513	0.82738336
31	H	3.09977275	-5.68960884	-0.60400144
32	H	5.22560854	-5.05969626	-1.87488871
33	N	2.89798203	-3.76972345	0.15256277
34	C	3.36487947	-2.51348365	0.22937882
35	N	3.76972345	2.89798203	0.15256277
36	C	2.51348365	3.36487947	0.22937882
37	N	-2.89798203	3.76972345	0.15256277
38	C	-3.36487947	2.51348365	0.22937882
39	N	-3.76972345	-2.89798203	0.15256277
40	C	-2.51348365	-3.36487947	0.22937882
41	O	-4.47767812	-1.91631247	2.52147817
42	O	-3.10894353	-0.46400722	1.01157099
43	O	0.46400722	-3.10894353	1.01157099
44	O	1.91631247	-4.47767812	2.52147817
45	O	4.47767812	1.91631247	2.52147817
46	O	3.10894353	0.46400722	1.01157099
47	O	-0.46400722	3.10894353	1.01157099
48	O	-1.91631247	4.47767812	2.52147817
49	S	-4.30541537	-1.27385839	1.22979301
50	S	1.27385839	-4.30541537	1.22979301
51	S	4.30541537	1.27385839	1.22979301
52	S	-1.27385839	4.30541537	1.22979301
53	H	-4.92835577	1.17353136	-0.41779773
54	H	-1.17353136	-4.92835577	-0.41779773
55	H	4.92835577	-1.17353136	-0.41779773
56	H	1.17353136	4.92835577	-0.41779773

5pyrazine/SO3 (-9938.5)

1	C	1.75259399	-6.61743604	-0.63910662
2	C	3.72378310	-4.77323970	-0.52032182
3	C	2.91304611	-6.72641012	-1.40485135
4	H	0.93955351	-7.33663879	-0.64887734
5	N	3.89845893	-5.81572019	-1.34982747
6	O	5.46258868	-1.14030633	2.64368459
7	H	3.04899291	-7.57288982	-2.07588884
8	H	0.26794989	4.47419698	0.91851229
9	C	-5.75197434	-3.71171613	-0.63910662
10	C	-5.49701542	-4.84904653	-1.40485135
11	H	-6.68722012	-3.16071456	-0.64887734

12	C	-3.38890847	-5.01654037	-0.52032182
13	N	-4.32638852	-5.50481114	-1.34982747
14	H	-6.26005559	-5.23991623	-2.07588884
15	O	0.60353697	-5.54760460	2.64368459
16	H	4.33801526	1.12776742	0.91851229
17	C	-5.30750964	4.32346932	-0.63910662
18	C	-6.31038848	3.72953455	-1.40485135
19	H	-5.07248284	5.38320976	-0.64887734
20	C	-5.81824372	1.67284725	-0.52032182
21	N	-6.57231408	2.41355980	-1.34982747
22	H	-6.91792004	4.33444350	-2.07588884
23	O	-5.08958232	-2.28830186	2.64368459
24	H	2.41309099	-3.77719839	0.91851229
25	C	2.47175299	6.38376712	-0.63910662
26	C	1.59698085	7.15402564	-1.40485135
27	H	3.55225332	6.48772116	-0.64887734
28	C	-0.20696391	6.05041683	-0.52032182
29	N	0.26447504	6.99647313	-1.34982747
30	H	1.98454588	7.91874964	-2.07588884
31	O	-3.74907183	4.13335627	2.64368459
32	H	-2.84664301	-3.46220440	0.91851229
33	C	6.83513700	-0.37808426	-0.63910662
34	C	5.69033299	2.06651599	-0.52032182
35	C	7.29737692	0.69189645	-1.40485135
36	H	7.26789613	-1.37357757	-0.64887734
37	N	6.73576864	1.91049840	-1.34982747
38	O	2.77252850	4.84285653	2.64368459
39	H	8.14443684	0.55961292	-2.07588884
40	H	-4.17241313	1.63743839	0.91851229
41	N	-4.56924668	3.55094940	0.17574710
42	C	-4.79568880	2.23026010	0.25599619
43	N	-4.78912845	-3.24830811	0.17574710
44	C	-3.60305273	-3.87178281	0.25599619
45	N	1.60940252	-5.55851422	0.17574710
46	C	2.56887975	-4.62315347	0.25599619
47	N	5.78379390	-0.18704260	0.17574710
48	C	5.19070773	1.01451684	0.25599619
49	N	1.96517869	5.44291553	0.17574710
50	C	0.63915406	5.25015935	0.25599619
51	S	3.21507700	4.35368255	1.34970055
52	S	5.13411159	-1.71235805	1.34970055
53	S	-0.04202154	-5.41197802	1.34970055
54	S	-5.16008234	-1.63242832	1.34970055
55	S	-3.14708472	4.40308183	1.34970055
56	O	5.99927000	-2.76349797	0.81065112
57	O	3.73408075	-1.73307897	0.93241625
58	O	-0.49436164	-4.08687269	0.93241625
59	O	-0.77436637	-6.55961266	0.81065112
60	O	-4.03961304	-0.79274725	0.93241625
61	O	-6.47785473	-1.29056561	0.81065112
62	O	-3.22916803	5.76199924	0.81065112
63	O	-2.00225652	3.59692794	0.93241625
64	O	4.48211913	4.85167699	0.81065112
65	O	2.80215046	3.01577097	0.93241625
66	H	-6.03224539	0.61034212	-0.44783459
67	H	-1.28359649	5.92561237	-0.44783459
68	H	5.23893913	3.05188773	-0.44783459
69	H	4.52143894	-4.03944202	-0.44783459
70	H	-2.44453619	-5.54840020	-0.44783459

6pyrazine/SO3 (-11924.4)

1	C	-7.49575012	2.94460671	-0.37536104
2	C	-5.43337344	4.66725160	-0.64617674
3	C	-7.62348904	4.14625239	-1.07287151
4	H	-8.29950402	2.22908152	-0.23219907
5	N	-6.60235700	5.00677404	-1.21346067
6	O	-4.08742822	-4.20082505	2.47464844
7	H	-8.57520632	4.41780564	-1.52643795
8	H	-4.32070139	3.18071032	0.50981398
9	C	-6.29797927	-5.01920667	-0.37536104
10	C	-7.40250443	-4.52900898	-1.07287151
11	H	-6.08019324	-6.07304057	-0.23219907
12	C	-6.75864516	-2.37181363	-0.64617674
13	N	-7.63717201	-3.21442187	-1.21346067
14	H	-8.11353507	-5.21744368	-1.52643795
15	O	1.59430710	-5.64022921	2.47464844
16	H	0.59422524	5.33219233	0.50981398
17	C	1.19777084	-7.96381338	-0.37536104
18	C	0.22098462	-8.67526138	-1.07287151
19	H	2.21931080	-8.30212207	-0.23219907
20	C	-1.32527172	-7.03906523	-0.64617674
21	N	-1.03481501	-8.22119591	-1.21346067
22	H	0.46167124	-9.63524932	-1.52643795
23	O	5.68173533	-1.43940415	2.47464844
24	H	4.91492663	2.15148201	0.50981398
25	C	7.49575012	-2.94460671	-0.37536104
26	C	7.62348904	-4.14625239	-1.07287151
27	H	8.29950402	-2.22908152	-0.23219907
28	C	5.43337344	-4.66725160	-0.64617674
29	N	6.60235700	-5.00677404	-1.21346067
30	H	8.57520632	-4.41780564	-1.52643795
31	O	4.08742822	4.20082505	2.47464844
32	H	4.32070139	-3.18071032	0.50981398
33	C	6.29797927	5.01920667	-0.37536104
34	C	7.40250443	4.52900898	-1.07287151
35	H	6.08019324	6.07304057	-0.23219907
36	C	6.75864516	2.37181363	-0.64617674
37	N	7.63717201	3.21442187	-1.21346067
38	H	8.11353507	5.21744368	-1.52643795
39	O	-1.59430710	5.64022921	2.47464844
40	H	-0.59422524	-5.33219233	0.50981398
41	C	-1.19777084	7.96381338	-0.37536104
42	C	1.32527172	7.03906523	-0.64617674
43	C	-0.22098462	8.67526138	-1.07287151
44	H	-2.21931080	8.30212207	-0.23219907
45	N	1.03481501	8.22119591	-1.21346067
46	O	-5.68173533	1.43940415	2.47464844
47	H	-0.46167124	9.63524932	-1.52643795
48	H	-4.91492663	-2.15148201	0.50981398
49	O	7.45618953	-0.39017878	1.04369525
50	O	5.06774368	-0.27014350	0.34895141
51	O	4.06599949	6.26216016	1.04369525
52	O	2.76782298	4.25372301	0.34895141
53	O	-3.39019004	6.65233894	1.04369525
54	O	-2.29992070	4.52386652	0.34895141
55	O	-7.45618953	0.39017878	1.04369525
56	O	-5.06774368	0.27014350	0.34895141
57	O	-2.76782298	-4.25372301	0.34895141
58	O	-4.06599949	-6.26216016	1.04369525
59	O	3.39019004	-6.65233894	1.04369525

60	O	2.29992070	-4.52386652	0.34895141
61	S	2.27545629	-5.71672262	1.19467706
62	S	6.08855515	-0.88775836	1.19467706
63	S	3.81309886	4.82896425	1.19467706
64	S	-2.27545629	5.71672262	1.19467706
65	S	-6.08855515	0.88775836	1.19467706
66	S	-3.81309886	-4.82896425	1.19467706
67	N	6.30783141	-2.63303964	0.16822456
68	C	5.26310672	-3.46758096	0.05340407
69	N	0.87363648	-6.77926207	0.16822456
70	C	-0.37145984	-6.29177460	0.05340407
71	N	-5.43419493	-4.14622242	0.16822456
72	C	-5.63456656	-2.82419364	0.05340407
73	N	-6.30783141	2.63303964	0.16822456
74	C	-5.26310672	3.46758096	0.05340407
75	N	-0.87363648	6.77926207	0.16822456
76	C	0.37145984	6.29177460	0.05340407
77	N	5.43419493	4.14622242	0.16822456
78	C	5.63456656	2.82419364	0.05340407
79	H	2.34267187	6.67064768	-0.72690786
80	H	6.94828629	1.30651048	-0.72690786
81	H	4.60561441	-5.36413719	-0.72690786
82	H	-2.34267187	-6.67064768	-0.72690786
83	H	-6.94828629	-1.30651048	-0.72690786
84	H	-4.60561441	5.36413719	-0.72690786
